# Cross-sectional and longitudinal analyses of the association between lung function and exercise capacity in healthy Norwegian men

**DOI:** 10.1186/s12890-018-0655-z

**Published:** 2018-07-18

**Authors:** Amir Farkhooy, Johan Bodegård, Jan Erik Erikssen, Christer Janson, Hans Hedenström, Knut Stavem, Andrei Malinovschi

**Affiliations:** 1 Department of Medical Sciences, Clinical Physiology, Uppsala University Hospital, SE-751 85 Uppsala, Sweden; 2 Department of Medical Sciences: Respiratory, Allergy and Sleep Research, Uppsala University, Uppsala, Sweden; 3 Department of Cardiology, Oslo University Hospital, Ullevaal, Norway; 4 Faculty of Medicine, University of Oslo, Oslo, Norway; 5 Institute of Clinical Medicine, University of Oslo, Lørenskog, Norway; 6 Department of Pulmonary Medicine, Medical Division, Akershus University Hospital, Lørenskog, Norway; 7 Health Services Research Unit, Akershus University Hospital, Lørenskog, Norway

## Abstract

**Background:**

It is widely accepted that exercise capacity in healthy individuals is limited by the cardiac function, while the respiratory system is considered oversized. Although there is physiological, age-related decline in both lung function and physical capacity, the association between decline in lung function and decline in exercise capacity is little studied. Therefore, we examined the longitudinal association between lung function indices and exercise capacity, assessed by the total amount of work performed on a standardized incremental test, in a cohort of middle-aged men.

**Methods:**

A total of 745 men between 40 and 59 years were examined using spirometry and standardized bicycle exercise ECG test within “The Oslo Ischemia Study,” at two time points: once during 1972–1975, and again, approximately 16 years later, during 1989–1990. The subjects exercise capacity was assessed as physical fitness i.e. the total bicycle work (in Joules) at all workloads divided by bodyweight (in kg).

**Results:**

Higher FEV_1_, FVC and PEF values related to higher physical fitness at both baseline and follow-up (all *p* values < 0.05). Higher explanatory values were found at follow-up than baseline for FEV_1_ (r^2^ = 0.16 vs. r^2^ = 0.03), FVC (r^2^ = 0.14 vs. r^2^ = 0.03) and PEF (r^2^ = 0.13 vs. r^2^ = 0.02). No significant correlations were found between decline in physical fitness and declines in FEV_1_, FVC or PEF.

**Conclusions:**

A weak association between lung function indices and exercise capacity, assessed through physical fitness, was found in middle-aged, healthy men. This association was strengthened with increasing age, suggesting a larger role for lung function in limiting exercise capacity among elderly subjects. However, decline in physical fitness over time was not related to decline in lung function.

## Background

The amount of oxygen consumed during exercise is dictated by the quantity of oxygenated blood distributed by the heart and the working muscle’s ability to take up the oxygen within that blood [1]. Thus, it is generally accepted that exercise capacity in healthy individuals is principally limited by maximum cardiac output [2, 3]. In contrast, the respiratory system is considered oversized in both respiratory volume and diffusing capacity, and is therefore believed not to be the limiting factor of maximum exercise capacity in healthy, non-endurance athletes [4]. Impaired lung function restricts the exercise capacity in patients with pulmonary disease [5, 6]. Although there is a physiological decline in lung function parameters with age [7], the association between age-related decline of lung function and decline in exercise capacity is little studied [8].

In healthy aging, there is a steady deterioration of the dynamic lung volumes. Both forced expiratory volume in one second (FEV_1_) and forced vital capacity (FVC) decline with age, and the flow-volume curve may change shape and become more similar to the curve in patients with chronic obstructive lung disease (COPD) [9, 10]. Normal aging of the lung can mimic the development of COPD in more ways than one [11]. The age-related loss of elastic tissue in the lung parenchyma exposes the airways to dynamic collapse during expiration causing a “pseudo-obstruction” that may be indistinguishable from true obstruction when only FEV_1_ is studied. In addition, with age, both the residual volume and the closing volume increase and alveolar walls disappear, producing a situation that has been termed “senile emphysema” [12]. The prevalence of dyspnoea increases with age in people not suspected of having lung disease, and physiological decline in lung function is believed to plays a role in the limitation of physical function in natural aging [13]. However, most studies investigating the relationship between declining lung function parameters and reduced maximum exercise capacity have been performed on elderly populations and/or with a cross-sectional study design [14–16].

To our knowledge, the impact of normal age-related decrease in lung function parameters on maximum exercise capacity in healthy middle-aged and young people has not previously been examined in a longitudinal study. Therefore, we wanted to investigate whether lung function indices were associated with maximum exercise capacity, assessed through physical fitness, in middle-aged, healthy subjects. Further, we sought to explore the relationship between age-related decline of lung function parameters and decrease of exercise capacity over time.

## Methods

### Subjects

The present analysis is based on data from a cardiovascular observational study, “The Oslo Ischemia Study,” in which men aged 40–59 years were recruited from five companies/governmental institutions in Oslo during the years 1972–1975. Of the 2341 apparently healthy men who were eligible and invited, 2014 men (86%) consented to participate. The participants had to be free from known or suspected heart disease, hypertension, diabetes mellitus, malignancy, advanced pulmonary, renal, or liver disease and should have no locomotor activity limitation. Further details about selection procedures and exclusion criteria have been presented elsewhere [17, 18]. The subjects underwent a clinical examination survey including questionnaires, assessment of cardiovascular risk factors, chest x-ray, dynamic spirometry and symptom-limited exercise test. The survey was repeated in 1989–1990 [19].

**Fig. 1:**
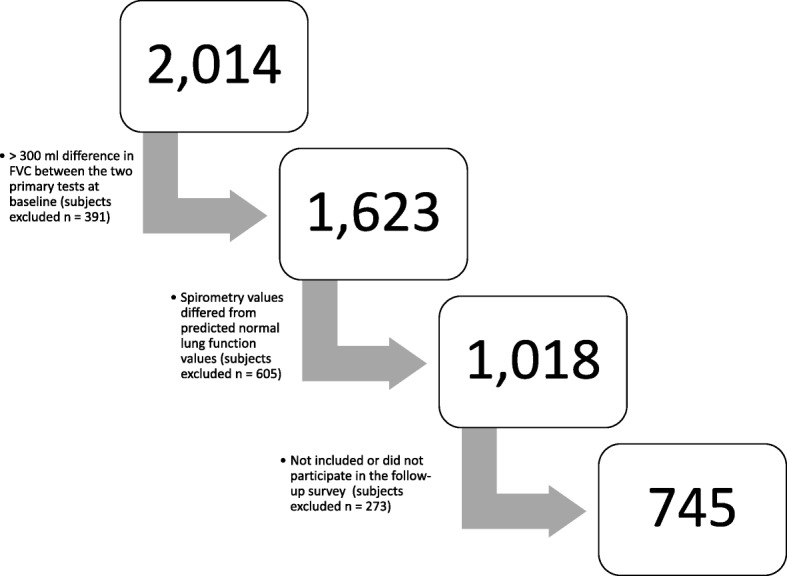
Survey flowchart regarding participants in the present study. Numbers in the text on the left side of the arrows represent excluded subjects due to respective criterion

Of the 2014 subjects enrolled at the baseline survey, 391 were excluded due to lack of spirometry or unsatisfactory quality of the lung function test (as outlined below). Furthermore, 605 subjects were excluded as their lung function values at the baseline survey differed from the predicted normal values, as described in greater detail below. The survey was repeated in 1989–1990, and a total of 273 subjects did not participate in the follow-up survey or were not included (a total of 12 subjects did not perform either exercise test or lung function testing). The remaining 745 subjects, with lung function and exercise capacity data from both surveys, were included in the current study (Fig. 1).

In 1972, no institutional or regional review board existed in Norway. Hence, no formal institutional approval for the investigation protocol could be obtained. However, the survey protocol was circulated among prominent physicians at two hospitals in Oslo, who commented on the protocol at an ad hoc meeting. All subjects gave their verbal informed consent before inclusion both at baseline and follow-up survey. Both study protocols underwent ethical assessments retrospectively and were approved by the regional committee for medical and health research ethics in Norway (REK nr. 188/89)*.* Nevertheless, written consent were gathered for the surviving cohort at 2007.

### Spirometry

At the baseline examination, FVC and FEV_1_ were measured with a calibrated Bernstein spirometer, using a standardized procedure [20]. After one trial test, FVC and FEV_1_ values were recorded from two successive maximum expiratory manoeuvres, corrected for body temperature and ambient pressure and saturated with water vapour, based on daily room temperature measurements and an assumption of atmospheric pressure of 760 mmHg. Originally, only the mean FEV_1_ and FVC values were recorded. To obtain the maximum of the two tests, the original spirograms and recorded values for both manoeuvres were retrieved in 2001 [21]. In order to increase the reliability of the data, as the original dataset was obtained before criteria for standardization were available, only subjects with < 0.3 L difference between the two FVC tests (*n* = 1625) were included, as previously described [22]. Additionally, in order to limit the present analyses to healthy individuals, only subjects with normal lung function values, defined as a FEV_1_/FVC ratio ≥ 0.7 and a FEV_1_ value greater than or equal to 80% of predicted, according to Norwegian reference values [23], were included in the current analysis. During the follow-up examination, a Vitalograph spirometer was used, with a similar protocol for the procedure. Peak expiratory flow (PEF) measurements were performed with a Wright’s peak flow meter, noting the mean value of the last two out of at least three tests.

### Exercise test

All participants performed a standardized bicycle exercise ECG test and were examined by the same physician, as previously described elsewhere [24]. The initial workload was 100 W for 6 min and then increased by 50 W every 6 min. The exercise test was continued until a heart rate of at least 90% of maximum predicted heart rate was reached, unless specific symptoms or signs necessitated premature termination. If an individual seemed physically fit despite reaching 90% of maximum predicted heart rate + 10 beats per minute at the end of one load, he was encouraged to continue as long as possible at the next load, i.e., at most an additional 6 min at a higher load. Exercise testing was repeated within 2 weeks in 130 of the participants and showed high reproducibility for heart rates and working capacity between the two tests, within ±5% in 90% of the men, and within ±10% in all of them [19, 25]. Exercise capacity, measured through physical fitness, was defined as the total bicycle work per unit of weight and calculated as the sum of work (in Joules) at all workloads divided by bodyweight (in kg).

### Anthropometric data

Height and weight were recorded at both the baseline and the follow-up visits.

### Questionnaire data

The subjects’ smoking habits (smoker/non-smoker) and exercise routines were recorded at the baseline visits. The subjects were divided into three groups based on their self-reported physical activity, as follows: 1) no existing exercise habits, 2) non-exhausting activity once in a while, and 3) routinely undertaking physical activity, from medium exhaustion at least five times per week up to competitive sports.

### Statistical analysis

Statistics were generated using computer software programs (STATA 12.1, StataCorp, College Station, TX, USA). Means ± standard deviations (SDs) were used to present descriptive statistics. A simple linear regression model was used to analyse the correlation between lung function parameters and variables relating to physical fitness. Only absolute values (L, L/min or kJ/kg) were used in all association analysis. These relations were tested for consistency at the baseline visit in a multiple linear regression model that included besides lung function parameters, age, height, exercise habits and current smoking, which are known as determinants of lung function and/or exercise capacity. A similar model at the follow-up visit included age (defined as age at start-up + 16 years, the median-follow-up time) and height, in addition to the lung function parameters. The longitudinal analysis on the change in lung function and physical fitness over time was done by means of simple linear regression.

The residuals in the regression models were checked for non-normality using plots versus fitted values and the dependent variables and appeared as normally distributed. A *p* value < 0.05, using two-sided tests, was considered statistically significant.

## Results

### Population characteristics

Subject’s characteristics for the whole group at inclusion are presented in Table 1. Lung function parameters at baseline and follow-up surveys are presented in Table 2. There was a significant decrease of lung function indices, in absolute values, and of physical fitness, between the baseline and follow-up surveys (Table 2).

**Table 1:** Subject characteristics at the baseline survey

	At baseline
Number of subjects	745
Age (years)	48.5 ± 5.3
Current smoker	245 (34.1%)
Height (cm)	176.4 ± 6.0
Weight (kg)	76.6 ± 9.3
BMI (kg/m^2^)	24.6 ± 2.6
Systolic BP (mmHg)	128.8 ± 16.7
Diastolic BP (mmHg)	86.4 ± 10.1
MAP (mmHg)	100.7 ± 11.8
Self-reported physical activity	
*No physical activity routine*	77 (10.3%)
*Low physical activity routine*	548 (73.6%)
*High physical activity routine*	120 (16.1%)

Legend: Values presented as mean (SD) or N (%). *BMI* body mass index, *BP* blood pressure, *MAP* mean arterial pressure

**Table 2:** Lung function and physical fitness at baseline and follow-up, *n* = 745

	Baseline visit		Follow-up visit		*p* value*
	Absolute values	% predicted	Absolute values	% predicted	
FEV_1_ (L)	3.8 ± 0.5	95.0 ± 9.8	3.5 ± 0.7	97.5 ± 16.3	< 0.001
FVC (L)	4.7 ± 0.7	95.6 ± 10.1	4.4 ± 0.7	92.8 ± 11.1	< 0.001
PEF (L/min)	558.4 ± 65.5	92.3 ± 10.2	544.7 ± 72.4	90.5 ± 12.0	< 0.001
Physical fitness (kJ/kg)	2.1 ± 0.8	–	1.02 ± 0.6	–	< 0.001

Legend: Values presented as mean ± SD. *FEV*_1_ forced expiratory volume in 1 s; *FVC* forced vital capacity, *PEF* peak expiratory flow. * *p* value for paired t-test for absolute values

### Physical fitness in relation to spirometry indices at baseline visit

**Fig. 2:**
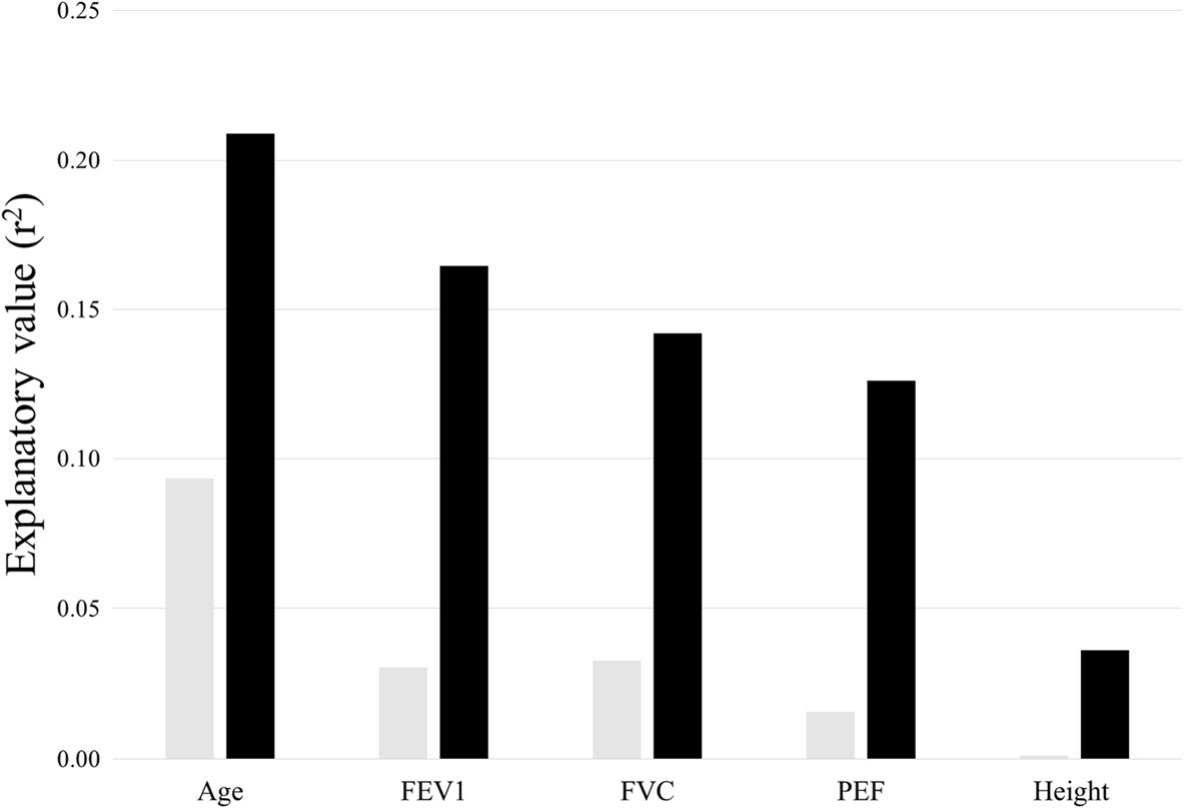
Comparison between surveys of explanatory values for physical fitness. Legend: Explanatory value (expressed in r^2^ value from a simple linear regression model) of each of the investigated parameters for physical fitness at baseline (grey) and follow-up (black)

A significant association of higher degree of self-reported physical activity, FEV_1_, FVC and PEF with higher objectively assessed physical fitness was found, as was a significant association of decreasing physical fitness with higher age (Fig. 2) and current smoking. The strongest correlation with physical fitness was found for subjects’ self-reported physical activities, followed by age. All three lung function parameters showed significant correlation with physical fitness (Fig. 2, Table 3).

**Table 3:** Regression coefficients (95% CI) of lung function for physical fitness at baseline (*n* = 745)

	Unadjusted	Adjusted^a^
FEV_1_ (per L)	0.26 (0.16, 0.37)	0.19 (0.08, 0.31)
FVC (per L)	0.22 (0.13, 0.31)	0.18 (0.08, 0.29)
PEF (per 100 L/min)	0.16 (0.07, 0.25)	0.09 (0.04, 0.17)

Legend: ^a^ adjusted for age, height, current smoking and physical activity habits

The relation with the three different lung function parameters was consistent also in multiple regression models after adjusting for age, height, weight, physical activity, and smoking (Table 3). FEV_1_ and FVC remained significantly related to physical fitness in a regression model containing all three lung function parameters, even after adjusting for age, height, physical activity and smoking (data not shown).

### Physical fitness in relation to spirometry indices at follow-up visit

In the follow-up survey, the strongest correlation with physical fitness was found for subject age, followed by FEV_1_ (Fig. 2). Significant associations of higher FEV_1_, FVC and PEF with higher physical fitness were found (Fig. 2, Table 4), as was as a significant association of lower physical fitness with higher age (Fig. 2).

**Table 4:** Regression coefficients (95% CI) of lung function for physical fitness at follow-up (*n* = 745)

	Unadjusted	Adjusted^a^
FEV_1_ (per L)	0.38 (0.32, 0.44)	0.25 (0.18, 0.32)
FVC (per L)	0.30 (0.25, 0.35)	0.20 (0.14, 0.27)
PEF (per 100 L/min)	0.28 (0.23, 0.33)	0.17 (0.12, 0.22)

Legend: ^a^ adjusted for age and height

Age and FEV_1_ had a higher explanatory value for a subject’s physical fitness at follow-up than at baseline (Fig. 2).

The relations with FEV_1_, FVC and PEF (all *p* <  0.001) were consistent also in multiple regression models after adjusting for age and height (Table 4). A similar model, where all three lung function parameters were inserted concomitantly, yielded PEF as the sole lung function parameter associated with physical fitness (p <  0.001), while no significant relations were found with FEV_1_ (*p* = 0.21) or FVC (p = 0.21).

### Decline in physical fitness in relation to decline in lung function or lung function at baseline

No significant correlation was found between decline in physical fitness and decline in FEV_1_ (*p* = 0.12), decline in FVC (*p* = 0.80), or decline in PEF (*p* = 0.78) when a simple linear regression model was used. A similar linear regression model with decline of physical fitness as outcome yielded neither baseline FEV_1_ (*p* = 0.22) nor baseline FVC (*p* = 0.36) as significant predictors. On the other hand, a significant negative correlation was found between decline in physical fitness and baseline PEF (r^2^ = 0.01, *p* = 0.02).

## Discussion

In the present study, we found that lung function indices obtained through dynamic spirometry (i.e., FEV_1_, FVC and PEF) were associated with exercise capacity, assessed through objectively measured physical fitness, in middle-aged, healthy men. This association was seen in cross-sectional analyses at both baseline and follow-up, approximately 16 years later, and, in fact, the relation to lung function seemed to increase over time. However, decline in physical fitness over time was not related to decline in lung function.

Numerous studies have explored the physiological changes in the respiratory system during aging [11, 26], but the relationship between physiological decline in lung function and declining exercise capacity has not been fully understood. Other studies investigating the impact of aging and exercise capacity have had a cross-sectional study design [27] and/or predominantly examined the relationship between cardiac function and exercise capacity [28]. To our knowledge, this is the first longitudinal study investigating the relationship between spirometric parameters and exercise capacity in healthy, middle-aged subjects.

As expected, our study subjects displayed physiological decline of lung function parameters over time. The decline of physical fitness by almost 50% constitutes a higher reduction in exercise capacity over time than in other similar studies [29]. We hypothesize that this is due to the exercise testing protocol used in the survey. Modern protocols uses 1–2-min incremental intervals, while we used 6-min intervals, a protocol more similar to steady-state exercise testing. This was the protocol of choice at the time of the baseline study. On the other hand, exercise capacity was presented as the cumulative workload divided by weight in the present study. Hence, an increase in weight over time would lead to lower calculated exercise capacity. This does differ from similar studies using treadmill exercise tests, in which bodyweight is not considered in calculating peak exercise capacity (in those protocols, exercise capacity is often converted to metabolic equivalent or is expressed as percent predicted for subject age). Another explanation might be development of cardiovascular or muscular limitations over time which were not quantified.

All lung function indices included in the study correlated significantly with physical fitness both at baseline and in the follow-up survey. All three investigated parameters, i.e., FEV_1_, FVC and PEF, behaved in the same manner, although they reflect different aspects. FEV_1_ is more closely related to airway obstruction, FVC to lung volumes and PEF to obstruction and muscular strength [30]. Given that the healthy respiratory system is oversized, one would expect to observe a relationship between lung function parameters and exercise capacity only when the dimensions of the respiratory system are reduced below a threshold value which represents the lower limit of normal lung function. However, there was a relationship between exercise capacity and lung function even at baseline, when the subjects were presumed healthy and had a normal lung function. The notion that lung function influences exercise capacity in healthy individuals is supported by previous animal study of Kirkton et al. [31], as they demonstrated that relatively larger lungs are required for increased endurance capacity in rats. Moreover, this relationship was strengthened at the follow-up survey, which was reflected in larger variation in lung function values at follow-up, with decreased values in some of the subjects. This may partly be related to a loss of lung elastic recoil in aging, which is associated with a reduction in the expiratory boundary of the maximal flow-volume envelope [32].

We could not find any association between decline in exercise capacity and decline in lung function. This might be attributed to the changes in exercise capacity being larger than the changes in lung function, and therefore the cardiovascular and muscular limitation of function over time may become more important for determination of physical fitness. The lack of association could also be referred to the fact that the pulmonary capacity is moving from a state of “overcapacity” in younger age to a weak limiting factor at older age. In such a situation, the decline in lung function is not expected to be associated with the decline in exercise capacity. Moreover, the design of our study with a rigorous selection of individuals with only normal spirometry values may also have limited the magnitude of changes in lung function. Furthermore, two different spirometry equipment were used between the surveys which could have an implication of the obtained results. However, we believe that exclusion of subjects with spirometry values below the normal range helped to ensure the accuracy of our original data gathered in the 1970s.

It could be discussed if there is a causal relation between loss of lung function and loss of physical fitness and the direction of this relation. One might contemplate that physiological decline in lung function with ageing may result in impaired physical fitness. However it could also be argued that decreased physical activity may result in accelerated loss in lung function, as suggested by a recent hypothesis article by Hopkinson and Polkey [33]. However, most of these studies cited in the article had primarily used questionnaire data on physical activity and there were no objective measures of physical fitness.

A strength of our study is the large number of participants and a follow-up period extending over 15 years. Some limitations of the study should be mentioned. The study included only male subjects, and spirometry was performed according to earlier and less rigorous standards than those used nowadays. Furthermore, we lack data on physical activity and smoking habits on follow-up survey, which could not be adjusted for at the follow-up visit, and therefore the adjusted models at baseline and follow-up visit differ. An additional limitation of the study is the use of a different protocol than those currently used for assessing exercise capacity, and a lack of normal values for physical fitness from other populations. The selection of subjects with normal lung function may be regarded as a strength, as we probably excluded subjects with possible respiratory disease. However, this may have contributed to a lower variability of the lung function variables in the baseline analysis. We did not have access to information regarding any comorbidities in the form of cardiovascular or neuromuscular limitations which might have developed during the follow-up period. The information regarding comorbidity could explain the relatively large decline in physical fitness between the surveys, which may have masked an effect of declining lung function. The information on physical activity at baseline was self-reported and this information is known to be inferior to objective measurements [34].

## Conclusions

In this study, lung function was significantly associated with physical fitness in healthy, middle-aged men both at baseline and follow-up surveys. Furthermore, our data suggests an increasing association between FEV_1_ and physical fitness with age, indicating that natural decline in lung functions may play a more essential role in the limitation of physical function in elderly. This finding might contribute to the understanding of the physiology of exercise and determinants of exercise capacity.

## Abbreviations

COPD: Chronic obstructive lung disease
FEV_1_: Forced expiratory volume in one second)
FVC: Forced vital capacity
PEF: Peak expiratory flow
